# The Relationship between Semantic Joke and Idiom Comprehension in Adolescents with Autism Spectrum Disorder

**DOI:** 10.3390/brainsci13060935

**Published:** 2023-06-09

**Authors:** Bat-el Yankovitz, Anat Kasirer, Nira Mashal

**Affiliations:** 1 The Faculty of Education, Bar-Ilan University, Ramat Gan 5290002, Israel; nmashal2@gmail.com; 2 Levinsky-Wingate Academic Center, Tel-Aviv 61480, Israel

**Keywords:** autism, humor, semantic joke, idiom, theory of mind

## Abstract

Semantic jokes involve resolving an incongruity emerging from wordplay or from a violation of world knowledge. The research has shown individuals with autism spectrum disorder (ASD) demonstrate a lower performance on humor tasks involving social situations; however, less is known about their semantic joke comprehension. This study examines semantic joke comprehension among adolescents with ASD and its possible relationship to vocabulary size, theory of mind (ToM), and idiom comprehension. Thirty-two adolescents with ASD and 32 typically developed (TD) peers participated. Semantic joke comprehension was assessed via a multiple-choice questionnaire and time-limited computer program. Vocabulary, ToM abilities, and idiom comprehension were also tested. The results reveal that adolescents with ASD are as fast in processing semantic jokes as their age- and vocabulary-matched TD peers, but less accurate. Age and idiom comprehension significantly contributed to semantic joke comprehension among both groups. As semantic joke comprehension is based on incongruity resolution, the greater difficulties in comprehension among the adolescents with ASD may have been due to deficits in simultaneously retaining two alternative interpretations and selecting the relevant one (and not due reduced ToM abilities). Similar to the TD group, semantic joke comprehension among the ASD group appeared to be more developed with age.

## 1. Introduction

Humor comprehension is a mental process that emerges from resolving incongruities [1,2,3]. One type of incongruity occurs in a semantic joke when a “punchline” is inconsistent with its contextual setup, leading to a mismatch with one’s previous expectation [4]. Understanding semantic jokes necessitates resolving incongruities that emerge from a violation of world knowledge, for example, *“Where’s the place with the cheapest rent?…The prison*” (adapted from [4]). Feelings of amusement and pleasure are elicited when a new piece of information causes a shift in understanding from a primary obvious script to a secondary or opposing script [5]. Another type of semantic joke relies on wordplay or puns, often exploiting multiple meanings of words or phrases, resulting in a surprising new meaning of the words used (e.g., “Two cannibals are eating a clown. One says to the other, Does this taste funny to you?”, derived from [6].

Two leading theories are often used to explain the processes involved in humor comprehension: the incongruity-resolution [7] and the comprehension–elaboration theories [8]. For the former, the cognitive processing of humor is conceptualized as a two-step model: an incongruity-detection stage followed by an incongruity-resolution stage. This theory posits that, to resolve incongruity issues, they must be removed. In the comprehension-elaboration theory, humor comprehension involves encoding a stimulus event based on its features using a schema already stored in one’s memory, as well as creating inferences based on the information relevant to understanding the event. Elaboration, on the other hand, involves forming a second schema by making conscious inferences about features not explicitly mentioned during the previous comprehension stage. This theory posits that the activation of the second schema allows the simultaneous existence of two incompatible interpretations. Both theories attempt to explain the tension elicited from an interpretation that does not match the straightforward meaning supplied by the set-up.

Delving more deeply into the subject, while neural activity can already activate upon encountering even the first few words of a joke, this activation changes as a joke is ultimately comprehended. The path of this neuronal activity is complex and enlists several networks: frontal and temporal brain regions to process the joke’s semantic information, and the subcortical and mesolimbic system to enjoy the reward that the joke’s resolution produces [9]. For example, an fMRI study found that a joke’s punchline activated language and semantic neural networks associated with incongruity detection and resolution [10]. These networks included the inferior frontal gyrus (IFG), middle temporal gyrus (MTG), superior temporal gyrus (STG), superior frontal gyrus (SFG), and inferior parietal lobule (IPL). The experience of amusement resulted in greater activation in reward regions, including the amygdala, nucleus accumbens, midbrain (VTA), and hypothalamus; furthermore, greater subjective funniness ratings were associated with increased activation in dopaminergic reward regions [10]. Thus, when a punchline is fully understood, the brain releases neurotransmitters, such as dopamine, shifting the focus of the brain’s response to its pleasure-and-reward center, evoking the hedonic aspects of humor [9]. A recent meta-analysis [11] confirmed the involvement of the IFG and middle and superior temporal gyri in humor comprehension regardless of stimulus type (visual or verbal, although activation was also observed in right homologues regions as well). These brain regions are involved in language and semantic integration processes required for most humor tasks.

Adolescence is characterized by changes in the frontal cortex [12] coupled with neurobiological processes, including greater cognitive function, verbal, and social behavior [13]. Executive functioning, such as working memory, shifting abilities, and mental flexibility, has been linked to frontal lobe development. Thus, executive functioning abilities that develop during adolescence may affect humor comprehension at this age. Similarly, changes in the structure of the brain are associated with vocabulary expansion during childhood and adolescence [14].

As for the response elicited by humorous stimuli, neural activation has been primarily observed in the medial prefrontal cortex, bilateral amygdala, ventral anterior cingulate cortex, and thalamus, corresponding to the emotional (amusement) response provoked by the unexpected “twist” in meaning and resolution of incongruity embedded within the humorous stimulus. Moreover, utilizing humorous stimuli based on theory of mind (ToM)—the ability to think about and act on information about one’s own and others mental states [15], such as jokes or cartoons in which incongruity arises from processing the players’ mental states [16,17,18,19]—studies have found activation mainly located in the medial prefrontal cortex and temporo-parietal junction. Although the findings of neuroimaging studies of humor processing can differ depending on the research task, stimulus type, and modality utilized, many researchers approach humor as a multifaceted function involving both a cognitive and affective processing stage (e.g., [16,20,21,22]).

The understanding of humor develops over the life course, playing an important role in social interactions and the formation of strong interpersonal connections [23,24]. The development of humor processing in most children with typical development (TD) increases with age. At the ages of 2–3 years, a child with TD already creates situations of incongruity in their own mind in a symbolic game with objects [25], even before they have developed the ability to understand another person’s mind. Around the age 8 years old, children with typical development can understand when a speaker says one thing but means another. At the ages of 10–14 years old, TD children can understand verbal jokes that include incongruity and complex lexical structures [25,26]. Humor plays a significant role, especially in adolescence, as it contributes to social acceptance and creating friendships. Age, indeed, plays an important role in understanding figurative language, as the development of advanced language skills and competencies facilitates the processing of complex expressions that require distinguishing between literal and figurative meanings (what we say vs. what we mean) [27]. Humor understanding thus develops over years and peaks in adolescence [25,26].

Accumulating evidence suggests that children with autism spectrum disorder (ASD) experience difficulties understanding non-literal language (of which humor is one subtype), including irony [28,29,30], metaphors [31,32,33], and idioms [32,34,35]. The evidence also suggests they lean towards literal interpretations of idioms instead of their lexicalized idiomatic meaning [32,36] and show difficulties in understanding and enjoying jokes, especially those that require considering people’s (false) mental states [37]. Asperger [38] was one of the first to describe this tendency among autistic individuals to fail to comprehend jokes or appreciate humor in general. Subsequently, Wu et al. [39] showed that adolescents with ASD had specific challenges resolving jokes involving incongruity, compared to the controls, and preferred nonsense jokes using homophones that create double meanings over jokes involving logical reasoning.

Several theories attempted to explain the source of the observed difficulties among those with ASD in understanding humor. Several scholars argue that theory of mind, a known core deficit in autism [40,41], is necessary for humor comprehension [42]. A recent study demonstrated that ToM skills (assessed by the Strange Stories task) predicted comprehension of mental jokes, but not phonological-based jokes, in older neurotypical adults [43]. Furthermore, a study using cartoons with and without ToM content found that neurotypical individuals with higher autistic traits failed to comprehend humorous cartoons, only when ToM was necessary for understanding them [44]. Among those with ASD, evidence has suggested that ToM skills are linked to comprehending jokes requiring social-situational understanding [15,37,45].

Non-literal language comprehension, in which one must fill the gap between literal and intended figurative meanings, has also been linked to mentalizing ability (e.g., [46,47]). Specifically, first-order ToM ability has been associated with metaphor comprehension, while second-order ToM skills have been linked to irony comprehension [48]. Consistent with this finding, idiom comprehension has been found to be associated with ToM ability in children with ASD, but not in children with TD [49]. One form of assessing mentalizing ability is using the Hinting test [29,50,51] that assesses the ability to understand the intensions of others. Indeed, irony understanding has been linked to Hinting test understanding in adolescents with ASD [29]. Although the present study focuses on semantic jokes that do not involve social-situational understanding, it explores whether semantic jokes (as manifestations of non-literal language) may be linked to mentalizing abilities, as was found in irony comprehension [29].

Alternative sources for the humor comprehension differences observed in ASD are proposed. One interpretation suggests they stem from difficulties in core language skills (e.g., vocabulary, syntax, and semantics) critical to the comprehension of figurative language [52]. Another interpretation is the weak central coherence (WCC) theory [53,54,55] that posits a processing style of focusing on details rather than the “whole picture.” Instead of perceiving the humor’s message level, individuals with ASD tend to focus on joke’s setup details that are irrelevant to the main message [15,56,57]. Others suggest the difficulties are sourced in executive function characteristic of children with ASD [58], such as challenges in working memory, shifting abilities, mental flexibility, and selective attention, all of which are involved in humor comprehension [15]. Indeed, a recent study [35] showed that ToM ability (as assessed by the Hinting test) and mental flexibility predicted the comprehension of ironic comic strips beyond group affiliation (TD or ASD) among adolescents aged 10–16 years old. Thus, various factors that typically characterize the ASD phenotype may affect joke comprehension [59,60].

To further explore how individuals with ASD may process semantic jokes differently, and to assess to what extent joke comprehension processing is difficult, accuracy and reaction time can be useful measures of the underlying cognitive processing stage. However, few studies have measured the reaction time to humorous stimuli among those with ASD. One study, conducted via computer, asked participants to decide whether cartoons with or without ToM content were funny [44]. While the study finding did not achieve significance, participants with high autistic traits showed longer reaction times for all cartoons, regardless of ToM content, and exhibited poorer humor comprehension on cartoons relying on ToM. Thiébaut et al. [61] also found a trend toward slower reaction times for participants with ASD, compared to neurotypical participants, when presented with a humor comprehension task (faux pas vs. non-faux pas cartoons). While a significant group by cartoon-type interaction was not found, participants with ASD reacted more slowly to both types of cartoons. Although suggesting a tendency toward slower reaction times and reduced accuracy among those with ASD or high autistic traits, cartoons were the only medium of humor stimuli tested, leaving a knowledge gap as to whether verbal semantic jokes might be processed more slowly or less accurately.

Moreover, the existing literature on this topic is sparse and inconclusive and has primarily focused on either adults [44,61] or children [62,63]. In the present study, semantic joke comprehension is measured utilizing two different tasks: a multiple-choice questionnaire and a computerized time-limited task. The multiple-choice questionnaire is a well-established measure of semantic joke comprehension by tasking participants to choose a punchline to complete given lines of a text by selecting one from among a few types of endings: funny, straightforward (not funny), or nonsensical [64,65]. The funny endings were based on wordplay and a violation of expectations (violation of world knowledge). The second semantic joke comprehension task was a time-limited computerized test that examined three types of utterances: semantic jokes (based on wordplay), literal sentences, and nonsense sentences [66]. Idiom comprehension was tested utilizing a multiple-choice questionnaire. The relationship between the comprehension of idioms and semantic jokes was thus examined among adolescents with ASD for the first time.

The aims of the present study are twofold: (1) to examine semantic joke understanding among adolescents with ASD compared to age- and vocabulary-matched TD controls, and (2) to examine the contribution of various abilities (vocabulary, ToM, and idiom comprehension) to semantic joke comprehension among adolescents with ASD and TD. We hypothesized that the adolescent TD group would score higher than the ASD group on the semantic joke comprehension questionnaire [57] and respond more accurately to semantic jokes on the computerized task, with no difference in reaction times between groups [44,61]. We also hypothesized no group differences in the reaction times to nonsense sentences [39,67]. Finally, we hypothesized idiom understanding and age would contribute to humor comprehension in both groups [15,27].

## 2. Materials and Methods

### 2.1. Participants

G*power software was used to determine a priori the sample size. For the primary analyses of a mixed-design ANOVA with repeated measures (3 × 2) and an effect size of 0.30 (small–medium), α error = 0.05, and power = 0.80: the total sample size required was 62 participants. To increase power and sensitivity, the present study included 64 participants, of whom 42 were male, 22 were females, and whose ages were between 11 and 16 years old (*M* = 13.64, *SD* = 1.09). The adolescents, all native Hebrew speakers, right- handed (not left-handed), were divided into two groups: those with a diagnosis of ASD and those with TD. All participants had normal or corrected-to-normal vision. The ASD group comprised 32 high-functioning adolescents (22 males; 10 females) aged 12–15 years old (*M* = 13.69, *SD* = 0.96). The TD group was comprised of 32 adolescents (20 males: 12 females) aged 11–16 years old (*M* = 13.59, *SD* = 1.21). No statistical differences were found between the two groups in gender distribution or age (χ^2^ (1) = 0.28, *p* = 0.599, t(62) = 0.34, *p* = 0.734, d = 0.09, respectively) (see Table 1). ASD diagnosis was previously determined by a psychiatrist according to the Diagnostic and Statistical Manual of Mental Disorders DSM-5 [68] criteria. Participants with either neurological impairments, focal neurological signs, or significant sensory impairments were not included in the study.

The recruitment of participants was conducted in accordance with institutional research guidelines and protocol approval was received by the Israeli Ministry of Education and the ethic committee of Bar-Ilan University. Letters were sent to parents about the study, requesting consent for their child’s participation; children whose parents provided signed informed consent were asked to participate. Adolescents with ASD were recruited from integrated classes within elementary and junior high schools, while those with TD were mostly recruited from junior high schools. Some participants were recruited by friends. Schools were from the same socioeconomic and sociocultural levels.

### 2.2. Tests

Various tests were used to assess participants’ vocabulary, theory of mind abilities, idiom comprehension, and semantic joke comprehension (see Table 1).

#### 2.2.1. Vocabulary

Vocabulary knowledge was examined using the vocabulary subtest of the Wechsler Intelligence Scale for Children—Hebrew (WISC-IV^HEB^ [69]). In this task, the participants were asked to provide definitions for the 28 words they heard. Scores ranged from 0–70.

#### 2.2.2. Theory of Mind (ToM)

ToM ability was assessed using the Hinting test. This test measures the comprehension of others’ intentions [51]. The task was originally designed by Corcoran et al. [50] and was professionally translated into Hebrew for a previous study [29]. The participants were requested to create ten brief stories depicting interactions between two individuals. Each story concluded with one of the speakers subtly hinting at something. Following the story, the participants were asked to discern the true intentions of the speaker: in other words, what the speaker actually meant. For instance, a question could be: “Rebecca mentioned to her father, ‘I love animals, especially dogs,’ as her birthday drew near. What does Rebecca truly imply with this statement?”. A correct answer, which accurately describes the speaker’s implicit intention, was awarded two points. In case the participants were unable to answer, they were provided with additional information. An example of such information could be: “Rebecca further asks, ‘Is the pet shop open on my birthday?’ What does Rebecca want her dad to do?”. A correct response given after the supplementary information was provided earned one point. If participants again failed to infer the intended meaning, they scored zero for the item. Total scores ranged from 0–20.

#### 2.2.3. Idiom Comprehension

This multiple-choice questionnaire examined comprehension of idioms [32]. Participants were presented with 20 idioms in Hebrew with plausible literal interpretations (e.g., he got cold feet). Each idiom was followed by four interpretations: a correct idiomatic interpretation, a literal interpretation of the idiomatic expression, a literal distracter related to or repeating the verb of the idiom, and an unrelated interpretation. Participants were instructed to read each idiom carefully and choose the correct interpretation. Each correct idiomatic interpretation received one point and total scores ranged from 0–20.

As seen in Table 1, groups did not significantly differ by gender, age, or vocabulary; however, the TD group scored higher than the ASD group in idiom comprehension and ToM.

#### 2.2.4. Semantic Jokes

##### Semantic Joke Comprehension: Multiple-Choice Questionnaire

This questionnaire examined the comprehension of semantic jokes and was based on the humor subtest of the Assessment of Pragmatic Abilities and Cognitive Substrates (APACS). The APACS was originally designed by Arcara and Bambini [64] to examine pragmatic language. This test was subsequently translated into Hebrew, modified to overcome cultural differences, and validated for those age 16 years and over (Cronbach’s alpha = 0.78) [70]. Participants were provided with short vignettes to read and asked to choose a funny ending from among three options: straightforward, unrelated, and humorous endings. For example: Mrs. Rossi visits a friend of hers. Upon seeing a beautiful piece of antique furniture, she exclaimed: “What a splendid piece! When does it date back to?” Her friend replied… (1. “To when we used to have money” (funny ending = correct); 2. “To the eighteenth century” (straightforward ending = incorrect); 3. “Woodworm is such a problem” (unrelated ending = incorrect)). The funny endings were based on two types of semantic jokes: those based on wordplay or on the violation of world knowledge.

The original APACS humor subtest included seven vignettes; however, after translation into Hebrew, two were removed due to the humorous punchline being “lost in translation”. To increase the number of the items in the questionnaire, 12 additional vignettes were composed in Hebrew by the study authors, and a pre-test was conducted with a separate group of participants to evaluate the humorous endings. Twenty TD individuals (10 females and 10 males; *M*_age_ = 23.75, *SD* = 4.99) participated in the pre-test. Participants were asked to rate the degree to which they found the ending of each vignette amusing on a scale of 1 (not amusing at all) to 5 (very amusing). Five items whose mean level of amusement was higher than three (*M* = 3.39, *SD* = 0.43; range = 3.2–4) were added to the questionnaire. Thus, the revised humor questionnaire contained 10 vignettes and total scores ranged from 0–10. Participant scores for the five original vignettes of Arcara and Bambini (2016) [64] and the five vignettes created for the present study were highly correlated, *r*(62) = 0.75, *p* < 0.001. Internal consistency (Cronbach’s alpha) for all 10 vignettes was high, α = 0.78.

##### Semantic Joke Comprehension: Computerized Experiment

This task examined reaction times and accuracy in processing semantic jokes. The stimulus pool consisted of 90 Hebrew sentences of 3 types: 30 semantic jokes, 30 literal sentences, and 30 nonsense sentences. Semantic jokes (e.g., The beggar asked the ice cream seller for some money. His response was quite cold) were constructed such that the last word was polysemic, i.e., having several meanings (e.g., cold), and evoked two associations: 1. the contextually relevant meaning derived from the setup content (unfriendly) and 2. the contextually irrelevant meaning (low temperature). Thus, while reading the humorous setup, the reader was biased toward choosing the contextually relevant “unfriendly” meaning while simultaneously ignoring the irrelevant “low temperature” meaning. Because both associations were activated, the reader felt enjoyment in the joke’s resolution. In contrast, literal sentences had only one meaning (e.g., Ron bought groceries at the supermarket. He bought dairy products and fruits) and nonsense sentences ended with an unrelated final word (e.g., Ashley came back from a bike ride. She drank chocolate and ate a wave) (see Table 2 for more examples).

Several pre-tests were performed to generate the stimuli. The first pre-test aimed at examining whether the semantic jokes’ final words evoked two intended associations: the contextually relevant meaning and the contextually irrelevant meaning linked to the sentence’s noun (either the subject or direct object). Ten participants (age range: 20–35 years old; *M* = 26.26; *SD* = 4.35) participated in the first pre-test and were presented with pairs of words: the sentences’ final words coupled with relevant (e.g., cold = unfriendly) and irrelevant (e.g., cold = low temperature) meanings. Participants were told: “Here are word pairs that may be: semantically related (clothes dress), a word and an adjective (safe travel), a familiar phrase (pay attention), or semantically unrelated (decision environment)”. Rate the strength of the association between each word pair on a scale from “1 = not related at all” to “7 = very related”. The results indicate that the strength of the association of contextually relevant meaning word pairs (*M* = 5.72, *SD* = 0.76) do not differ from the contextually irrelevant meaning word pairs (*M* = 6.01, *SD* = 0.69), *t*(29) = 1.81, *p* > 0.05.

The second pre-test was aimed at examining the extent to which the final word of each joke was surprising and enjoyable. Thirty participants (10 males) aged 18–34 years old (*M* = 27.4, *SD* = 5.42) participated in this pre-test. These participants did not take part in the first pre-test or in the live experiment and were asked to rate how surprising the last word in each sentence was on a scale from “1 = not at all surprising” to “7 = very surprising”. The participants were also asked to indicate to what extent the sentences were enjoyable for them on a scale from “1 = not at all enjoyable” to “7 = very enjoyable”. All items rated above 4 on each scale (surprising: *M* = 5.08, *SD* = 0.47, enjoyable: *M* = 5.01, *SD* = 0.59) confirmed their amusement from the semantic jokes.

In addition, analyses were performed to counterbalance conditions according to the final word’s syntactic category, frequency, and length. There were five types of syntactic categories: adjectives, nouns, verbs, words that were both nouns and adjectives, and words that were both verbs and adjectives. Word frequency was based on Internet norms data [71] (semantic jokes: *M* = 6235, *SD* = 14,607, literal: *M* = 6235, *SD* = 14,607, nonsense: *M* = 6367, *SD* = 16,139), and the length of the final word was also counterbalanced between conditions (semantic jokes: *M* = 4.50, *SD* = 1.54, literal: *M* = 4.47, *SD* = 1.25, nonsense: *M* = 4.50, *SD* = 1.38), *F*(2, 27) = 0.006, *p* = 0.99.

##### Procedure for the Computerized Semantic Joke Experiment

Participants sat in front of a computer screen at a comfortable viewing distance. Stimuli were displayed via SuperLab version 5.0 software on a laptop (Cedrus Corp., San Pedro, CA, USA). First, a fixation point appeared at the center of the screen for 2000 ms, and once the point disappeared, a sentence was presented in the center of the screen. Participants were asked to press the button when they finished reading the sentence. Then, a fixation point was presented for 250 ms, after which the target stimulus (the sentence’s final word) appeared in the center of the screen and remained there for 1000 ms. Participants were instructed to decide whether the final word made a logical ending to the sentence, indicating “yes” (by pressing the N keyboard key with their right finger) or “no” (by pressing the B keyboard key with their right finger). They were asked to make the semantic decision as accurately and as quickly as possible. The semantic jokes, literal sentences, and nonsense sentences were presented in a random order. Each session began with a practice session of nine trials not included in the live experimental session.

##### General Experimental Procedure

Parents provided signed informed consent for their child’s participation in the study, and after receiving an explanation of the study, adolescents agreeing to participate were enrolled. After completing tasks assessing vocabulary, idiom comprehension, and ToM, they performed the computerized task. Following a 10 min break, participants were then asked to complete the multi-choice joke questionnaire.

## 3. Results

We conducted Mauchly’s test to examine the sphericity assumption. For those cases in which the assumption of sphericity was rejected, we reported the adjusted degree of freedom (*df*) in decimal number format.

### 3.1. Semantic Joke Comprehension Questionnaire

To examine the possible differences between ASD and TD participants in semantic joke comprehension questionnaire scores, we conducted a *t*-test analysis for two independent samples. A significant difference between the two study groups was found, *t*(62) = 4.86, *p* < 0.001, indicating lower accuracy among the ASD (*M* = 5.94, *SD* = 2.51), compared to the TD, group (*M* = 8.63, *SD* = 1.86).

### 3.2. Computerized Semantic Joke Experiment

Performance on the computerized semantic joke experiment was analyzed by a two-way (2 × 3) mixed ANOVA. One analysis was performed on reaction times and the other on accuracy (percent correct responses). Independent variables were Group as the between-subjects factor (ASD, TD) and Stimuli Type as the within-subjects factor (semantic jokes, literal sentence, nonsense sentence) (see Table 3).

Reaction times: The main effect of Stimulus Type was significant, *F*(1.770, 109.749) = 49.21, *p* < 0.001, η_p_^2^ = 0.44. Pair-wise comparisons indicated faster reaction times for literal sentences compared to the semantic jokes or nonsense sentences (*ps* < 0.001). No significant difference was found in the reaction times for the semantic jokes and nonsense sentences (*p* = 0.375). No significant main effect of Group or interaction for Group and Stimulus Type were found (*F*(1, 62) = 2.32, *p* = 0.133, η_p_^2^ = 0.04 and *F*(2, 124) = 1.14, *p* = 0.324, η_p_^2^ = 0.02, respectively).

**Figure 1:**
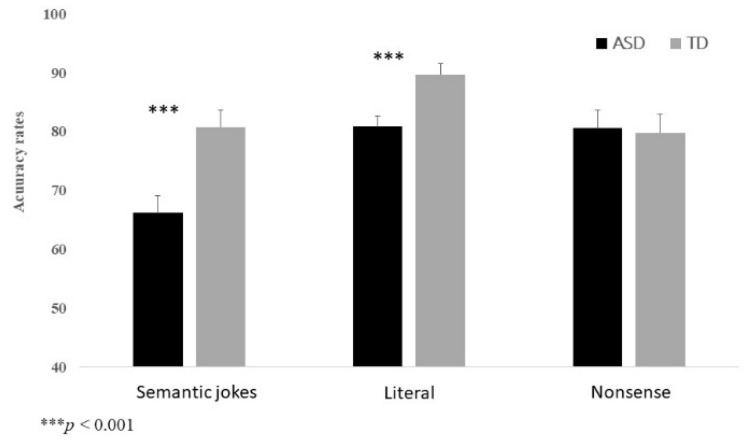
Percent of accuracy rates in the computerized semantic joke experiment by group and stimulus type.

Accuracy rate: The main effect of Group was significant, *F*(1, 62) = 11.43, *p* = 0.001, η_p_^2^ = 0.16. The results indicate that the accuracy rates in the computerized semantic joke experiment are significantly higher among the TD students compared to the ASD students. The main effect of Stimuli Type was significant, *F*(1.448, 89.765) = 4.44, *p* = 0.025, η_p_^2^ = 0.07. Pair-wise comparisons indicated that the accuracy rate was significantly higher for literal sentences compared to the semantic jokes (*p* < 0.001). No significant differences were found in the accuracy rates between nonsense sentences and jokes or literal sentences (*p* = 0.130 and *p* = 0.131, respectively). Finally, a significant interaction for Group and Time was found, *F*(2, 124) = 4.44, *p* = 0.014, η_p_^2^ = 0.07. Bonferroni analyses comparing the two study groups for each stimulus type indicated that, while significant differences were found between the two study groups in the accuracy rate for semantic jokes and literal sentences (*ps* < 0.001), no significant difference was found for the nonsense sentence type (*p* = 0.868). The results thus indicate that the accuracy rates for semantic jokes and literal sentences are significantly higher among the TD students compared to the ASD students (see Figure 1).

Speed–accuracy trade-off: Pearson’s correlations were conducted between the accuracy rates and reaction times for each study group and each condition, separately. The results indicate no significant correlations between the accuracy rates and reaction times for the computerized semantic joke experiment in both groups (Pearson’s coefficients between −0.31 and 0.02 and *p*-values between 0.082 and 0.898), indicating no speed accuracy trade-off in the computerized semantic joke experiment among both groups.

### 3.3. Regression Analysis

To examine our second hypothesis regarding the contribution of the participants’ gender, age, vocabulary, idiom comprehension, and ToM to their scores on the semantic joke questionnaire, a hierarchical regression analysis was conducted for each study group. In the first step of the regression model, the students’ background characteristics (gender, age) were entered in a step-wise manner. Only variables that contributed significantly to the explained variance (EPV) were entered (see Table 4). Scores on vocabulary, idiom comprehension, and ToM tests were then entered in a step-wise manner.

As Table 4 demonstrates, the participants’ age contributed significantly to the EPV of the semantic joke questionnaire scores for both the ASD and TD groups (12.3% and 12.8%, respectively). In the second step, the participants’ scores on the idiom comprehension test contributed significantly to the EPV of the semantic joke questionnaire scores for both groups (ASD: 28.6%, TD: 45.7%). The positive β coefficients indicated that as the participants’ ages and idiom test scores increased, the semantic joke comprehension increased, respectively. Vocabulary and ToM scores did not contribute significantly to the model and are therefore not shown in Table 4.

It should be noted that the contribution of the idiom comprehension test scores to the EPV of the semantic jokes questionnaire scores was almost twice as much among the TD students compared to the ASD students. In order to examine whether the grouping variable served as a mediating variable between the idiom test and semantic joke questionnaire scores, a moderation analysis using Process software was conducted [72]. The results indicate a significant interaction (*R*^2^ = 3.88%, *F*(1, 60) = 5.68, *p* = 0.020), meaning that the grouping variable served as a mediating variable between the idiom test scores and the semantic joke questionnaire scores.

## 4. Discussion

The present study investigated semantic joke processing among adolescents with ASD compared to age- and vocabulary-matched TD peers. Our results indicate that adolescents with ASD understand fewer semantic jokes than their TD peers, both via the multiple-choice questionnaire and a timed computerized task. Notwithstanding, participants with ASD were as fast as their TD peers in processing semantic jokes and literal sentences. Age and idiom comprehension contributed significantly to semantic joke understanding scores for both groups; however, idiom comprehension contributed twice as much to semantic joke understanding for the TD compared to the ASD group.

As expected, and consistent with the previous studies [15,63,73], participants with ASD demonstrated a reduced ability in both semantic joke comprehension tasks compared to their TD peers. Various studies point to difficulties in understanding humor among those with ASD, regardless of the type of task utilized, whether involving computers [43], questionnaires [39], or video clips [63]. Furthermore, previous evidence has suggested that individuals with ASD perform worse than the controls in understanding jokes that rely on ToM ability and understanding social situations (i.e., mental jokes) [44,61,74]. However, rather than using social scenarios relying on ToM abilities (mental jokes), in the present study, we used semantic jokes either based on wordplay that required suppressing irrelevant associations or based on the violation of expectations that required a resolution of world knowledge violations. For instance, in the computerized semantic joke task, two different associations were activated by each item’s final word: the contextually relevant meaning and the irrelevant association. Participants had to focus on the contextually relevant association and ignore the irrelevant one, in order to comprehend the joke. Indeed, several studies have indicated that people with ASD tend to have difficulty with verbal tasks that include solving ambiguous sentences (e.g., [75,76]) and that require using context in order to understand the text [77]. A possible explanation for our findings may be linked to the weak central coherence theory. As individuals with ASD tend to focus on small details, the ability to ignore the irrelevant association may pose an obstacle for correct joke interpretation among this population [37,57].

Another possible explanation for the reduced accuracy in understanding semantic jokes observed in the current study may be linked to an impaired ability to integrate upcoming information with the context. To understand semantic jokes that contain world knowledge violation, the reader must recognize the mismatch between the joke’s contextual setup (that builds expectations and anticipation for a certain input based on world knowledge) and the “punchline” (a new and unexpected piece of information). Thus, to recognize the incongruity of a joke the reader must integrate his background knowledge with the contextual setup. Several studies point to a reduced tendency for semantic integration in ASD [35,78], a deficit which may be critical mechanisms for joke comprehension. In line with this view, Marzi et al. (2023) [79] further suggested a reduced anticipatory use of contextual information to predict upcoming stimuli in ASD. According to the authors, because of a reduced integration of experience, it was more difficult for individuals with ASD to use past knowledge to generate predictions about the occurrence of new events. This may explain the reduced accuracy in recognizing a violation of world knowledge.

The current study also found no differences in reaction times between groups on the computerized semantic joke task. That said, the ASD group in our study tended to respond to the semantic jokes more slowly in comparison to the TD group (1424 vs. 1286 ms, respectively), despite the data not reaching statistical significance. This finding reflects the previous findings that also did not reach statistical significance [44,61] of a tendency toward slower processing times of semantic jokes among those with ASD compared to those with TD. Our study further expanded the existing ASD literature by examining RTs among participants with ASD who read verbal jokes and performed a semantic judgment task, whereas Aykan and Nalçacı’s [44] study had participants decide whether a cartoon was funny or not. Thus, it may be that when humor tasks are based on decoding pictorial information that involves social understanding, or are based on an incongruity resolution, a trend was seen in which those with ASD needed more time than the TD controls to process humor. However, further research is needed to determine whether this observation has true statistical significance.

It should also be noted that no trade-off between reaction times and accuracy was observed. It is possible that, as previously pointed out, individuals with ASD can enjoy (appreciate) a joke without necessarily understanding it [39]. Moreover, although high-school students with ASD have been found to exhibit less comprehension of all types of jokes than TD controls, they still have a sense of humor and report enjoyment from reading nonsense jokes. Indeed, unlike the semantic jokes and literal sentences, ASD participants processed the nonsense sentences as accurately and as rapidly as the TD group.

We also sought to identify the extent to which background characteristics (gender, age) as well as vocabulary size, ToM ability, and idiom comprehension scores contributed to predicting semantic joke comprehension (as assessed by the semantic joke questionnaire). Our findings indicate that age contributes to semantic joke comprehension in both study groups. It has been previously posited that, among TD individuals, verbal humor understanding develops between 10–14 years of age [25], at which point children can understand verbal jokes that include incongruity and complex lexical structures [25]. The current study participants were similarly aged (11–16 years old) and indeed age was found to significantly contribute to semantic joke understanding. To our knowledge, these findings are the first to indicate that age predicts semantic joke understanding among adolescents with ASD. Notwithstanding, more studies are needed to further examine the development of joke comprehension among those with ASD using variety of tasks and age ranges.

Our results also show that idiom comprehension is a significant predictor of semantic joke comprehension in both groups. This finding is not surprising, given that idiom understanding, similar to other components of figurative language, shares the needed ability to compute the nonliteral contextually relevant interpretation of an expression and to suppress the irrelevant literal interpretation [15,35,52]. Notably, in our study, idiom comprehension contributed to predicting semantic joke comprehension questionnaire scores almost twice as much among the adolescents with TD as compared to the ASD participants. Unlike the developmental course of figurative language (i.e., idioms and humor) among individuals with TD [25,80], figurative language development seemed to be delayed in ASD [81]. It has been purported that its development among individuals with ASD depends more on their unique cognitive profile, the features of which are thought to include weak central coherence [57], mind blindness [42], and impaired executive functions [60]. The current study’s findings thus indicate a connection between group membership and the comprehension of semantic jokes. Specifically, individuals with TD demonstrated a more advanced capacity to understand figurative language, including idioms. This enhanced ability, in turn, correlated with their higher proficiency in comprehending semantic jokes.

No contribution of ToM ability and vocabulary size to semantic joke understanding was found in the current study. While the ability to understand the intentions of the other is extremely important for humor comprehension [42], the current study’s humor was based on wordplay and a violation of expectations in which the reader had to focus on the relevant interpretation while suppressing the irrelevant association. According to the comprehension–elaboration theory [8], humor comprehension involves activating a schema already stored in the memory, followed by an elaboration stage that involves forming a second schema that allows the simultaneous activation of two incompatible interpretations. It is plausible that the participants with ASD were less efficient in forming or maintaining these two schemas concurrently, and this may have contributed to the reduced comprehension of semantic jokes found a72mong our ASD cohort [15,37].

There were study limitations that should be mentioned. The abilities we tested in addition to semantic joke comprehension (vocabulary size, idiom comprehension, ToM) were rather limited and need to be expanded to include a broader range of central coherence abilities and executive functions in order to determine their contribution to successful semantic joke comprehension [55]. Additionally, given the sparse research on the development of humor comprehension in ASD, future studies should test a broader age range and various types of humor (including mental jokes).

## 5. Conclusions

Our findings show that adolescents with ASD understand semantic jokes worse than their age- and vocabulary-matched TD peers across two tasks (a multiple-choice semantic joke questionnaire and a computerized semantic joke task). Idiom comprehension contributed significantly to predicting semantic joke comprehension (beyond vocabulary size and ToM ability) in both groups, but more so for the TD group. Thus, our findings show that reduced ToM abilities do not contribute to semantic joke comprehension. These findings suggest that one difficulty in semantic joke comprehension among adolescents with ASD may be related to a reduced ability to understand idioms, possibly due to challenges creating or maintaining two alternative interpretations and selecting the relevant one. However, similarly to the TD group, their understanding of semantic jokes becomes more developed with age.

## Figures and Tables

**Table 1:** Participant gender, age, and performance on assessments of abilities by group.

Background Characteristics	ASD *M* (*SD*)	TD *M* (*SD*)	*t*	*p*	Cohen’s *d*
Gender (Males/Females) ^!^	22/10	20/12	0.28	0.599	---
Age	13.69 (0.96)	13.59 (1.21)	0.34	0.734	0.09
Vocabulary	39.41 (5.70)	40.13 (7.68)	−0.42	0.672	0.11
Idiom Comprehension ^1^	14.03 (4.28)	18.69 (1.82)	5.66 ***	0.001	1.42
ToM ^2^	14.22 (3.02)	18.13 (1.72)	6.35 ***	0.001	1.59

^!^ Chi-squared analysis was conducted; ^1^ *df* = 41.855; ^2^ *df* = 49.124; *** *p* < 0.001.

**Table 2:** Examples of stimuli used in the computerized task.

Semantic Joke	Literal Sentence	Nonsense Sentence
I told Isaac the tailor a good joke. He was in stiches.	John was excited during the flight. He especially liked to fly above the clouds.	Sarah had a big test yesterday. She studied and during the test she had a lake.
The children waited a very long time at the airport before deciding to go home. Time did not fly.	The baby was very tired. His mother decided that now he should be put to sleep in bed.	Tammy went to the mall. She went into a jewelry store and bought a fly.
Bad traffic caused Taylor to stay home with her baby. It was crawling.	David went outside after the rain. He looked to the sky and saw a rainbow.	Romi decided to go jogging this morning. She ran down the street to the mustache.

Note: almost all of the original Hebrew semantic jokes did not translate meaningfully into English (i.e., retaining their humor); therefore, the examples provided in English are for illustrative purposes only.

**Table 3:** Mean (*SD*) reaction times and accuracy rates on the computerized semantic joke experiment by group and stimuli type.

Stimulus Type	ASD (*n* = 32)		TD (*n* = 32)		*F* Values		
	*M*	*SD*	*M*	*SD*	Group	Stimulus Type	Interaction
**Reaction Times**							
					2.32	49.21 ***	1.14
Semantic Jokes	1424.39	373.10	1286.22	363.62			
Literal	1307.96	341.65	1152.46	328.14			
Nonsense	1438.31	362.11	1332.11	377.29			
**Accuracy Rates**							
					11.43 ***	10.43 ***	4.44 *
Semantic Jokes	66.15	18.28	80.73	13.93			
Literal	80.83	9.27	89.69	10.82			
Nonsense	80.52	17.19	79.79	17.82			

* *p* < 0.05, *** *p* < 0.001.

**Table 4:** Hierarchical regression analyses for the humor comprehension questionnaire scores by participant characteristics for each study group.

Independent Variables	*B*	*SE.B*	*β*	*R^2^*	∆*R*^2^
**ASD (*n* = 32)**					
Step 1: Age	0.91	0.44	0.35 *	0.123 *	0.123 *
Step 2: Age	0.63	0.38	0.24		
Idioms	0.32	0.08	0.54 ***	0.409 ***	0.286 ***
**TD (*n* = 32)**					
Step 1: Age	0.55	0.26	0.36 *	0.128 *	0.128 *
Step 2: Age	0.09	0.20	0.06		
Idioms	0.76	0.13	0.74 ***	0.585 ***	0.457 ***

* *p* < 0.05, *** *p* < 0.001.

## Data Availability

The data presented in this study are available on request from the corresponding author.

## References

[1] McGhee P.E. (1976). Children’s Appreciation of Humor: A Test of the Cognitive Congruency Principle. Child Dev..

[2] Nomura R., Maruno S. (2011). Constructing a coactivation model for explaining humor elicitation. Psychology.

[3] Shultz T.R. (1972). The role of incongruity and resolution in children’s appreciation of cartoon humor. J. Exp. Child Psychol..

[4] Chang Y.T., Ku L.C., Wu C.L., Chen H.C. (2019). Event-related potential (ERP) evidence for the differential cognitive processing of semantic jokes and pun jokes. J. Cogn. Psychol..

[5] Masaeli B., Heidari-Shahreza M.A. (2016). A linguistic analysis of Persian online jokes in light of general theory of verbal humor. J. Appl. Linguist. Lang. Res..

[6] Martin R.A. (2007). The Psychology of Humor: An Integrative Approach.

[7] Suls J.M., Goldstein J.H., McGhee P.E. (1972). A two-stage model for the appreciation of jokes and cartoons: An information-processing analysis. The Psychology of Humor: Theoretical Perspectives and Empirical Issues.

[8] Wyer R.S., Collins J.E. (1992). A theory of humor elicitation. Psychol. Rev..

[9] Mobbs D., Greicius M.D., Abdel-Azim E., Menon V., Reiss A.L. (2003). Humor Modulates the Mesolimbic Reward Centers. Neuron.

[10] Shibata M., Terasawa Y., Umeda S. (2014). Integration of cognitive and affective networks in humor comprehension. Neuropsychologia.

[11] Farkas A.H., Trotti R.L., Edge E.A., Huang L.Y., Kasowski A., Thomas O.F., Sabatinelli D. (2021). Humor and emotion: Quantitative meta analyses of functional neuroimaging studies. Cortex.

[12] Blakemore S.J., Choudhury S. (2006). Development of the adolescent brain: Implications for executive function and social cognition. J. Child Psychol. Psychiatry.

[13] Yurgelun-Todd D. (2007). Emotional and cognitive changes during adolescence. Curr. Opin. Neurobiol..

[14] Breit M., Brunner M., Preckel F. (2020). General intelligence and specific cognitive abilities in adolescence: Tests of age differentiation, ability differentiation, and their interaction in two large samples. Dev. Psychol..

[15] Lyons V., Fitzgerald M. (2004). Humor in autism and Asperger syndrome. J. Autism Dev. Disord..

[16] Chan Y.C., Lavallee J.P. (2015). Temporo-parietal and frontoparietal lobe contributions to theory of mind and executive control: An fMRI study of verbal jokes. Front. Psychol..

[17] Feng S., Ye X., Mao L., Yue X. (2014). The activation of theory of mind network differentiates between point-to-self and point-to-other verbal jokes: An fMRI study. Neurosci. Lett..

[18] Marjoram D., Job D.E., Whalley H.C., Gountouna V.E., McIntosh A.M., Simonotto E., Cunningham-Owens D., Johnstone E.C., Lawrie S. (2006). A visual joke fMRI investigation into theory of mind and enhanced risk of schizophrenia. NeuroImage.

[19] Samson A.C., Zysset S., Huber O. (2008). Cognitive humor processing: Different logical mechanisms in nonverbal cartoons—An fMRI study. Soc. Neurosci..

[20] Campbell D.W., Wallace M.G., Modirrousta M., Polimeni J.O., McKeen N.A., Reiss J.P. (2015). The neural basis of humour comprehension and humour appreciation: The roles of the temporoparietal junction and superior frontal gyrus. Neuropsychologia.

[21] Chan Y.C., Chou T.L., Chen H.C., Yeh Y.C., Lavallee J.P., Liang K.C., Chang K.E. (2013). Towards a neural circuit model of verbal humor processing: An fMRI study of the neural substrates of incongruity detection and resolution. Neuroimage.

[22] Vrticka P., Black J.M., Reiss A.L. (2013). The neural basis of humour processing. Nat. Rev. Neurosci..

[23] Airenti G. (2016). Playing with expectations: A contextual view of humor development. Front. Psychol..

[24] Fraley B., Aron A. (2004). The effect a shared humorous experience on closeness in initial encounters. Pers. Relatsh..

[25] Semrud-Clikeman M., Glass K. (2010). The relation of humor and child development: Social, adaptive, and emotional aspects. J. Child Neurol..

[26] Weisfeld G.E. (1993). The adaptive value of humor and laughter. Ethol. Sociobiol..

[27] Bernicot J., Laval V., Chaminaud S. (2007). Nonliteral language forms in children: In what order are they acquired in pragmatics and metapragmatics?. J. Pragmat..

[28] MacKay G., Shaw A. (2004). A comparative study of figurative language in children with autistic spectrum disorders. Child Lang. Teach. Ther..

[29] Saban-Bezalel R., Dolfin D., Laor N., Mashal N. (2019). Irony comprehension and mentalizing ability in children with and without autism spectrum disorder. Res. Autism Spectr. Disord..

[30] Wang A.T., Lee S.S., Sigman M., Dapretto M. (2006). Neural basis of irony comprehension in children with autism: The role of prosody and context. Brain.

[31] Geurts B., Kissine M., van Tiel B., Morsanyi K., Byrne R.M.J. (2019). Pragmatic reasoning in autism. Thinking, Reasoning, and Decision Making in Autism.

[32] Mashal N., Kasirer A. (2011). Thinking maps enhance metaphoric competence in children with autism and learning disabilities. Res. Dev. Disabil..

[33] Morsanyi K., Stamenković D., Holyoak K.J. (2020). Metaphor processing in autism: A systematic review and meta-analysis. Dev. Rev..

[34] Saban-Bezalel R., Mashal N. (2019). Different factors predict idiom comprehension in children and adolescents with ASD and typical development. J. Autism Dev. Disord..

[35] Vulchanova M., Saldaña D., Chahboun S., Vulchanov V. (2015). Figurative language processing in atypical populations: The ASD perspective. Front. Hum. Neurosci..

[36] Kerbel D., Grunwell P. (1998). A study of idiom comprehension in children with semantic-pragmatic difficulties. Part II: Between-groups results and discussion. Int. J. Lang. Commun. Disord..

[37] Samson A.C., Hegenloh M. (2010). Stimulus characteristics affect humor processing in individuals with Asperger syndrome. J. Autism Dev. Disord..

[38] Asperger H. (1944). Die “Autistischen Psychopathen” im Kindesalter [Autistic psychopathy in childhood]. Arch. Psychiatr. Nervenkrankh..

[39] Wu C.L., Tseng L.P., An C.P., Chen H.C., Chan Y.C., Shih C.I., Zhuo S.L. (2014). Do individuals with autism lack a sense of humor? A study of humor comprehension, appreciation, and styles among high school students with autism. Res. Autism Spectr. Disord..

[40] Baron-Cohen S. (1988). Social and pragmatic deficits in autism: Cognitive or affective?. J. Autism Dev. Disord..

[41] Happé F. (1993). Communicative competence and theory of mind in autism: A test of relevance theory. Cognition.

[42] Howe N.E. (2002). The origin of humor. Med. Hypotheses.

[43] Bischetti L., Ceccato I., Lecce S., Cavallini E., Bambibi V. (2019). Pragmatics and theory of mind in older adults’ humor comprehension. Curr. Psychol..

[44] Aykan S., Nalçacı E. (2018). Assessing theory of mind by humor: The humor comprehension and appreciation test (ToM-HCAT). Front. Psychol..

[45] Jung W.E. (2003). The inner eye theory of laughter: Mindreader signals cooperator value. Evol. Psychol..

[46] Baron-Cohen S., Leslie A.M., Frith U. (1985). Does the autistic child have a ‘theory of mind’?. Cognition.

[47] Happé F., Winner E., Brownell H. (1998). The getting of wisdom: Theory of mind in old age. Dev. Psychol..

[48] Happé F.G.E. (1995). Understanding minds and metaphors: Insights from the study of figurative language in autism. Metaphor Symb. Act..

[49] Whyte E.M., Nelson K.E., Scherf K.S. (2014). Idiom, syntax, and advanced theory of mind abilities in children with autism spectrum disorders. J. Speech Lang. Hear. Res..

[50] Corcoran R., Mercer G., Frith C.D. (1995). Schizophrenia, symptomatology and social inference: Investigating “theory of mind’’ in people with schizophrenia. Schizophr. Res..

[51] Turner-Brown L.M., Perry T.D., Dichter G.S., Bodfish J.W., Penn D.L. (2008). Brief report: Feasibility of social cognition and interaction training for adults with high functioning autism. J. Autism Dev. Disord..

[52] Kalandadze T., Norbury C., Nærland T., Næss K.A.B. (2018). Figurative language comprehension in individuals with autism spectrum disorder: A meta-analytic review. Autism.

[53] Frith U. (1989). Autism: Explaining the Enigma.

[54] Happé F.G.E. (1997). Central coherence and theory of mind in autism: Reading homographs in context. Br. J. Dev. Psychol..

[55] Happé F., Frith U. (2006). The weak coherence account: Detail-focused cognitive style in autism spectrum disorders. J. Autism Dev. Disord..

[56] Samson A.C., Huber O., Ruch W. (2013). Seven decades after Hans Asperger’s observations: A comprehensive study of humor in individuals with Autism Spectrum Disorders. Humor.

[57] Wu C.L., Liu Y.R., Kuo C.C., Chen H.C., Chang Y.L. (2016). Effectiveness of humor training among adolescents with autism. Psychiatry Res..

[58] Van Eylen L., Boets B., Steyaert J., Evers K., Wagemans J., Noens I. (2011). Cognitive flexibility in autism spectrum disorder: Explaining the inconsistencies?. Res. Autism Spectr. Disord..

[59] Demetriou E.A., Lampit A., Quintana D.S., Naismith S.L., Song Y.J., Pye J.E., Guastella E.A. (2018). Autism spectrum disorders: A meta-analysis of executive function. Mol. Psychiatry.

[60] Emerich D.M., Creaghead N.A., Grether S.M., Murray D., Grasha C. (2003). The comprehension of humorous materials by adolescents with high-functioning autism and Asperger’s syndrome. J. Autism Dev. Disord..

[61] Thiébaut F.I., White S.J., Walsh A., Klargaard S.K., Wu H.C., Rees G., Burgess P.W. (2016). Does faux pas detection in adult autism reflect differences in social cognition or decision-making abilities?. J. Autism Dev. Disord..

[62] Gill C., White G., Allman T. (2011). Teaching lexical humor to children with autism. Stud. Lit. Lang..

[63] Purser H.R., Van Herwegen J., Ranzato E., Thomas M.S. (2021). The role of context in verbal humor processing in autism. J. Exp. Child Psychol..

[64] Arcara G., Bambini V. (2016). A test for the Assessment of Pragmatic Abilities and Cognitive Substrates (APACS): Normative data and psychometric properties. Front. Psychol..

[65] Shammi P., Stuss D.T. (2003). The effects of normal aging on humor appreciation. J. Int. Neuropsychol. Soc..

[66] Yankovitz B.E., Mashal N. (2020). Can brain stimulation improve semantic joke comprehension?. J. Cogn. Psychol..

[67] Weiss E.M., Gschaidbauer B.C., Samson A.C., Steinbäcker K., Fink A., Papousek I. (2013). From ice age to Madagascar: Appreciation of slapstick humor in children with Asperger’s syndrome. Humor.

[68] American Psychiatric Association [APA] (2013). Diagnostic and Statistical Manual of Mental Disorders.

[69] Wechsler D. (2003). Wechsler Intelligence Scale for Children: WISC-IV.

[70] Fussman S., Mashal N. (2022). Initial Validation for the Assessment of Pragmatic Abilities and Cognitive Substrates (APACS) Hebrew Battery in Adolescents and Young Adults With Typical Development. Front. Commun..

[71] Linzen T. (2009). Corpus of Blog Postings Collected from the Israblog Website.

[72] Hayes A.F. (2012). PROCESS: A Versatile Computational Tool for Observed Variable Mediation, Moderation, and Conditional Process Modeling [White Paper]. http://www.afhayes.com/public/process2012.pdf.

[73] Treichel N., Dukes D., Barisnikov K., Samson A.C. (2022). How cognitive, social, and emotional profiles impact humor appreciation: Sense of humor in autism spectrum disorder and Williams syndrome. Humor.

[74] Silva C., Da Fonseca D., Esteves F., Deruelle C. (2017). Seeing the Funny Side of Things: Humour Processing in Autism Spectrum Disorders. Res. Autism Spectr. Disord..

[75] Davidson M.M., Ellis Weismer S. (2017). Reading comprehension of ambiguous sentences by school-age children with autism spectrum disorder. Autism Res..

[76] Jolliffe T., Baron-Cohen S. (2001). A test of central coherence theory: Can adults with high-functioning autism or Asperger syndrome integrate fragments of an object?. Cogn. Neuropsychiatry.

[77] Van Deijck N.J.H. (2016). Resolving Homonyms in Context: Understanding Ambiguity for Autism Spectrum Disorder. Ph.D. Dissertation.

[78] Gold R., Faust M., Goldstein A. (2010). Semantic integration during metaphor comprehension in Asperger syndrome. Brain Lang..

[79] Marzi C., Narzisi A., Milone A., Masi G., Pirrelli V. (2022). Reading Behaviors through Patterns of Finger-Tracking in Italian Children with Autism Spectrum Disorder. Brain Sci..

[80] Cain K., Towse A.S., Knight R.S. (2009). The development of idiom comprehension: An investigation of semantic and contextual processing skills. J. Exp. Child Psychol..

[81] Rundblad G., Annaz D. (2010). The atypical development of metaphor and metonymy comprehension in children with autism. Autism.

